# Impairment of vocational activities and financial problems are frequent among German blood cancer survivors

**DOI:** 10.1038/s41598-023-50289-9

**Published:** 2023-12-21

**Authors:** Julia Baum, Hildegard Lax, Nils Lehmann, Anja Merkel-Jens, Dietrich W. Beelen, Karl-Heinz Jöckel, Ulrich Dührsen

**Affiliations:** 1 Klinik für Hämatologie, Universitätsklinikum Essen, Universität Duisburg-Essen, Hufelandstraße 55, 45147 Essen, Germany; 2 Institut für Medizinische Informatik, Biometrie und Epidemiologie, Universität Duisburg-Essen, Essen, Germany; 3 Klinik für Knochenmarktransplantation, Universitätsklinikum Essen, Universität Duisburg-Essen, Essen, Germany

**Keywords:** Diseases, Health care, Medical research, Oncology

## Abstract

Little is known about changes in the personal living conditions of long-term blood cancer survivors in Germany. To gather information about social relationships, work life, overall well-being, and religion, we performed a questionnaire-based retrospective study on 1551 survivors who had been on follow-up for ≥ 3 years (median, 9 years). Most survivors reported that marital status and relationships with relatives and friends remained constant before and after blood cancer. Vocational activities were temporarily impaired for 47.5%, with a median time of 11 months to return to work. More than a third of the patients (35.6%) discontinued work permanently, with disability and retirement pension rates of 7.9% and 38.1%, respectively, at the time of the survey. Financial problems due to reduced income were reported by 26.2%, in particular after relapse or allogeneic transplantation. Patient reports addressing their quality of life showed large variations. It was best in acute leukemia survivors without a history of allogeneic transplantation and worst in patients with myeloproliferative disorders. Religion tended to become more important after blood cancer. In conclusion, vocational impairment and financial problems are frequent among German blood cancer survivors. Efforts should be made at an early stage to reestablish the patients’ ability to work.

## Introduction

Malignancies of the blood-forming system are the fourth most common type of cancer in the western world^1,2^, with a continuously rising incidence during the past decades^3–5^. Due to improvements in diagnostic procedures, risk stratification, and treatment, the prognosis for most blood cancer subtypes has considerably improved, with long-term survival rates of 50–90%^6,7^.

The diagnosis of blood cancer is a turning point in the life of affected patients. Treatment tends to be long and intense, and may be complicated by life-threatening side effects. Relapses are frequent, in particular during the first 2 or 3 years^8–10^. Several studies have evaluated the impact of blood cancer on the survivors’ quality of life (reviewed in^11–14^), but little is known about other aspects of their personal life that are strongly influenced by the socio-economic conditions of the countries where they reside. Most available studies have focused on specific hematological diseases, specific treatments, and specific aspects of long-term survivorship. Relationships with family members and friends^15–19^ and return-to-work issues^20–27^ have been studied in several European countries, Australia, and the USA. Reports from the Netherlands provided detailed information about financial problems^28,29^. To our knowledge, there is only one study from Germany addressing personal living conditions. It was restricted to patients undergoing autologous blood stem cell transplantation for hematological malignancies or solid tumors and was published more than 20 years ago^30^. Since a decreasing minority of hematological diseases are treated by autologous transplantation, for the majority of German blood cancer survivors, information about personal living conditions is not available.

To gather information about blood cancer survivors in Germany, we launched the questionnaire-based ‘Aftercare in Blood Cancer Survivors’ (ABC) study. Its primary goal was to identify and compare follow-up institutions. To this end, blood cancer survivors from the University Hospital of Essen, the oldest and one of the largest comprehensive cancer centers in Germany, were contacted and asked to name the institutions currently providing follow-up care. Study design, patient recruitment, and types of follow-up institutions have been described^31^.

The study offered the opportunity to extend the survey to include aspects other than blood cancer follow-up institutions. One of the sections of the patient questionnaire focused on personal living conditions. The goal of this substudy was to gain information about the impact of blood cancer on the survivors’ relationships with family members and friends, their work life, their financial situation, their quality of life, and their attitude towards religion.

## Patients and methods

### Eligibility

The patient eligibility criteria have been previously described^31^. In brief, patients ≥ 18 years diagnosed with and/or treated for a hematological malignancy at the University Hospital of Essen were eligible for the study, provided that the interval between study inclusion and the date of diagnosis (for untreated patients) or the end of last treatment (for primary disease, relapse, or a second primary malignancy) was ≥ 3 years. In patients receiving continuous oral medication or low dose maintenance therapy after intensive induction, eligibility started 3 years after treatment initiation or end of induction, respectively. Because relapse tends to occur early in hematological malignancies^8–10^, initial follow-up is usually provided by the institution where the patient received treatment. Survivors without early relapse may subsequently migrate to other institutions. To be able to compare different institutions we limited the study to survivors with an interval since the last intense treatment of at least 3 years. Patients exclusively treated in childhood or adolescence were not eligible^31^. The term ‘blood cancer’ encompassed all types of hematological malignancies including leukemias, myelodysplastic syndromes, myeloproliferative diseases, lymphomas, multiple myeloma, and its precursor, monoclonal gammopathy of undetermined significance. In an effort to establish a manageable number of disease categories, characterized by similar treatment approaches and similar prognosis, the diseases were allocated to seven groups: monoclonal gammopathy of undetermined significance (MGUS), multiple myeloma (MM), indolent non-Hodgkin lymphoma including chronic lymphocytic leukemia (iNHL/CLL), myeloproliferative neoplasms including chronic myeloid leukemia (MPN/CML), myelodysplastic syndromes (MDS), aggressive non-Hodgkin or Hodgkin’s lymphoma (aNHL/HL), and acute myeloid or acute lymphoblastic leukemia (AML/ALL). Irrespective of the underlying disease, patients undergoing allogeneic transplantation were allocated to a separate group (AlloTx), because health issues arising ≥ 3 years after transplantation are more likely to be related to the procedure than to the disease^31^.

### Study design

The ABC study was an observational study performed from October 2013 to December 2016. It comprised a retrospective study and an 18-month prospective study^31^. It was performed in line with the principles of the Declaration of Helsinki and approved by the Ethics Committee of the University of Duisburg-Essen (February 17, 2014; no. 14-5692-BO). The present manuscript describes the results of a substudy within the retrospective part. All participating patients gave written informed consent.

In the retrospective part of the study, eligible patients were identified by hospital documents spanning the period from 1999 to 2010^31^. The patients were informed by mail about the purpose of the study and invited to complete a 118-item questionnaire specifically designed for the study. Twenty-one questions were related to personal living conditions (see Supplementary Information S1). Quality of life was assessed by the German versions of the EORTC QLQ C-30 and Hospital Anxiety and Depression Scale (HADS) questionnaires. Patients not responding within 4–6 weeks were contacted by mail again, and patients failing to respond to the second invitation were reminded by phone^31^.

### Statistical analysis

The analysis focused on three areas: social relationships (seven questions of the questionnaire related to marital status, family, friends and relatives), work life (11 questions related to employment, financial situation, and health insurance), and overall well-being and spirituality (two quality-of life questionnaires, one question related to religion). The analysis included comparisons of the period before and after blood cancer (social relationships, employment, health insurance, religion), comparisons of blood cancer subgroups (financial situation, quality of life), comparisons of survivors with or without disease relapse (financial situation), comparison of different age groups (employment, financial situation), and comparisons of different follow-up periods (4–5, 6–10, < 10 years; quality of life).

The statistical methods employed in the ABC study have been described before^31^. Frequencies are presented as numbers and compared using the chi^2^ test. Unless otherwise stated, percentages refer to the total number of patients, i.e., they are not corrected for missing data. Continuous data are presented as median, first and third quartile (interquartile range, IQR), compared using the Kruskal–Wallis test, and graphically displayed as box-whisker plots, diamonds representing means. All analyses are exploratory, assuming statistical significance at p ≤ 0.05.

The quality-of-life assessment included the broad domains global health, functioning (physical, role, cognitive, emotional, and social combined), and symptoms of the EORTC QLQ C-30 questionnaire and anxiety and depression of the HADS questionnaire. It was restricted to the time of the survey. The following transformations from the log-transform family were found to yield well normalized scales, suitable for analysis of variance (ANOVA) and co-variance (ANCOVA):

$$\begin{gathered} {\text{Y}} = {\text{A}} + {\text{B}}*{\text{ln}}\left( {{\text{C}} + {\text{D}}*{\text{X}}} \right) \hfill \\ {\text{X}} = {\text{global health scale }}\left( {{\text{EORTC QLQ C}} - {3}0} \right):{\text{ A}} = {5},{\text{ B}} = - {1},{\text{ C}} = {14}0,{\text{ D}} = - {1} \hfill \\ {\text{X}} = {\text{functional scale }}\left( {{\text{EORTC QLQ C}} - {3}0} \right):{\text{ A}} = {5},{\text{ B}} = - {1},{\text{ C}} = {12}0,{\text{ D}} = - {1} \hfill \\ {\text{X}} = {\text{symptom scale }}\left( {{\text{EORTC QLQ C}} - {3}0} \right):{\text{ A}} = 0,{\text{ B}} = {1},{\text{ C}} = {2}0,{\text{ D}} = {1} \hfill \\ {\text{X}} = {\text{anxiety scale }}\left( {{\text{HADS}}} \right):{\text{ A}} = 0,{\text{ B}} = {1},{\text{ C}} = {1}0,{\text{ D}} = {1} \hfill \\ {\text{X}} = {\text{depression scale }}\left( {{\text{HADS}}} \right):{\text{ A}} = 0,{\text{ B}} = {1},{\text{ C}} = {1},{\text{ D}} = {1} \hfill \\ \end{gathered}$$

Note that the sense of direction is maintained, i.e. with increasing X, Y also increases. To adjust the influence of ‘disease group’ (nominal, p value for group difference) and ‘time since last treatment or diagnosis’ (ordinal, p value for trend) on the quality-of-life scales for age and sex, the transformed scales were modelled using respective general linear models.

## Results

### Patients

Of 2555 patients contacted, 841 men and 710 women participated in the study^31^. The median age was 58 years (range, 23–91), the median time from diagnosis was 11 years (range, 3–41), and the median time from last treatment was 9 years (range, 3–36). The survivors were allocated to 7 groups of diseases not treated by allogeneic transplantation, and one allogeneic transplantation group comprising all 554 transplanted patients irrespective of the underlying disease (Table 1). The survivors were asked whether their present living conditions differed from those before the advent of blood cancer.

**Table 1:** Patient characteristics^31^.

Total number of patients	1551
No allogeneic transplantation—number of patients (% of total number)	997 (64.3%)
MGUS	19
MM	37
iNHL/CLL	264
MPN/CML	107
MDS	5
aNHL/HL	491
AML/ALL	74
Allogeneic transplantation^a^—number of patients (% of total number)	554 (35.7%)
Age at study entry—years, median (range)	57.6 (23.0–91.2)
Time from diagnosis—years, median (range)	10.5 (3.0–40.7)
Time from last treatment^b^—years, median (range)	8.9 (3.0–36.0)
Male—number of patients (% of total number)	841 (54.2%)
Female—number of patients (% of total number)	710 (45.8%)
Follow-up period—number of patients (% of total number)	
Year 4–5	292 (18.8%)
Year 6–10	528 (34.1%)
Year > 10	731 (47.1%)

*MGUS* monoclonal gammopathy of undetermined significance, *MM* multiple myeloma, *iNHL* indolent non-Hodgkin lymphoma, *CLL* chronic lymphocytic leukemia, *MPN* myeloproliferative neoplasm, *CML* chronic myeloid leukemia, *MDS* myelodysplastic syndrome, *aNHL* aggressive non-Hodgkin lymphoma, *HL* Hodgkin lymphoma, *AML* acute myeloid leukemia, *ALL* acute lymphoblastic leukemia.

^a^Allogeneic transplantation for MM, 9 patients; iNHL/CLL, 24; MPN/CML, 219; MDS, 40; aNHL/HL, 23; AML/ALL, 239.

^b^Time from last treatment in 1279 survivors receiving blood cancer treatment (82.5% of total number of survivors).

### Social relationships

#### Marital status

The number of survivors that were married (1118 [72.1%] versus 1089 survivors [70.2%]; p = 0.2505) or were living with a partner (1205 [77.7%] versus 1173 survivors [75.6%]; p = 0.1744) was similar before and after blood cancer. Between diagnosis and end of treatment, losing a partner was slightly more frequent than finding a new partner (53 versus 44 patients), the difference being fully accounted for by the patient’s partner’s death during the treatment period (9 patients). Between the end of treatment and the time of the survey, 83 survivors lost their partner (34 partners died) and 104 previously single survivors found a partner.

#### Family

In 710 of 984 survivors who reported to have lived with the same partner before and after blood cancer, the relationship was described as unchanged (72.2%). 209 survivors reported an improvement (21.2%) and 65 a deterioration (6.6%). Most survivors also had an unchanged relationship with their children. In 844 of 1054 survivors who had one or more children at the time of diagnosis, the relationship remained unchanged (80.1%), while an improvement was reported by 174 survivors (16.5%) and a deterioration by 36 (3.4%).

#### Friends and relatives

Irrespective of gender, the average number of social contacts was not affected by blood cancer. The median numbers of close friends (4; IQR, 2–6), relatives (5; IQR, 3–9), and children (2; IQR 1–2; analysis restricted to patients with children) were identical before and after the hematological malignancy (919–1201 responding survivors per question). The median numbers of close friends (3; IQR, 2–5), relatives (4; IQR, 2–6), and children (2; IQR, 1–2) seen at least once per month also remained unchanged.

### Work life

#### Employment

The time after the hematological malignancy was characterized by a statistically significant decrease in full-time employment (from 49.3 to 27.5%), with a concomitant increase in disability (from 2.2 to 7.9%) and retirement (from 15.2 to 38.1%). The decrease in full-time employment and the increase in disability and retirement were statistically significant for the entire population and the age groups between 40 and 65 years (Table 2). This was not fully accounted by the number of survivors reaching the official retirement age of 65 years, as it was also seen in younger age groups (Table 2). For example, the age group 40–49 years comprised 343 patients at the time of the blood cancer diagnosis and 274 at the time of the survey. Here, the full-time employment rate decreased from 61.5 to 48.2%, while the disability and retirement rates increased from 1.5 to 10.2% and from 1.5 to 9.9%, respectively. The non-employment rate (8.9% [134 of 1502 responding survivors] versus 9.1% [136/1488]; p = 0.8350) remained constant. It included survivors seeking work (2.4% [36/1502] versus 2.4% [35/1488]; p = 0.9361) and survivors taking care of household and family (6.5% [98/1502] versus 6.8% [101/1488]; p = 0.7730).

**Table 2:** Employment status at the time of blood cancer diagnosis and at the time of the survey.

Age (years)^a^	Number of survivors affected/responding (%)								
	Full-time employment			Disability			Retirement		
	Diagnosis	Survey	p^c^	Diagnosis	Survey	p^c^	Diagnosis	Survey	p^c^
18–19	7/68 (10.3%)	n.a.^b^	n.a.	0/68 (0.0%)	n.a.^b^	n.a.	0/68 (0.0%)	n.a.^b^	n.a.
20–29	99/173 (57.2%)	28/57 (49.1%)	0.2860	1/173 (0.6%)	1/57 (1.8%)	0.4068	1/173 (0.6%)	1/57 (1.8%)	0.4068
30–39	213/319 (66.8%)	73/124 (58.9%)	0.1186	6/319 (1.9%)	5/124 (4.0%)	0.1914	4/319 (1.3%)	2/124 (1.6%)	0.7692
40–49	211/343 (61.5%)	132/274 (48.2%)	0.0009	5/343 (1.5%)	28/274 (10.2%)	< 0.0001	5/343 (1.5%)	27/274 (9.9%)	< 0.0001
50–59	188/338 (55.6%)	146/400 (36.5%)	< 0.0001	16/338 (4.7%)	62/400 (15.5%)	< 0.0001	32/338 (9.5%)	57/400 (14.3%)	0.0468
60–65	33/130 (25.4%)	35/348 (10.1%)	< 0.0001	2/130 (1.5%)	24/348 (6.9%)	0.0215	66/130 (50.8%)	218/348 (62.6%)	0.0186
> 65	4/159 (2.5%)	4/316 (1.3%)	0.3178	3/159 (1.9%)	0/316 (0.0%)	0.0143	125/159 (78.6%)	274/316 (86.7%)	0.0232
Total	755/1530 (49.3%)	418/1519 (27.5%)	< 0.0001	33/1530 (2.2%)	120/1519 (7.9%)	< 0.0001	233/1530 (15.2%)	579/1519 (38.1%)	< 0.0001

^a^Age at the time of the blood cancer diagnosis or survey, respectively.

^b^No survivors below age 23 at the time of survey (see Table 1); *n.a.* not applicable.

^c^Chi^2^ test.

Of 966 responding patients in employment at the time of diagnosis, 163 (16.9%) reported no negative impact of blood cancer on their vocational activities, 459 (47.5%) reported temporary impairment lasting a median of 11 months (IQR, 6–12), and 344 (35.6%) reported permanent discontinuation. The latter group had not resumed their former profession for a median time period of 9 years (IQR, 6–13) at the time of the survey. There were statistically significant differences between different age groups (Table 3). While temporary impairment was most pronounced in young patients, permanent discontinuation of vocational activities was most frequently reported by survivors age 40–49 at the time of diagnosis.

**Table 3:** Impairment of vocational activities and financial problems associated with the diagnosis of blood cancer.

Age (years)^a^	Number of survivors affected/responding (%)		
	Vocational activities^b^		Financial problems^e^
	Temporary impairment^c^	Permanent discontinuation^d^	
18–19	26/39 (66.7%)	8/39 (20.5%)	13/68 (19.1%)
20–29	86/141 (61.0%)	32/141 (22.7%)	52/165 (31.5%)
30–39	130/256 (50.8%)	94/256 (36.7%)	129/303 (42.6%)
40–49	121/277 (43.7%)	113/277 (40.8%)	103/335 (30.7%)
50–59	82/214 (38.3%)	84/214 (39.3%)	70/316 (22.2%)
60–65	13/33 (39.4%)	12/33 (36.4%)	8/116 (6.8%)
> 65	4/6 (66.7%)	1/6 (16.7%)	2/136 (1.5%)
Total	459/966 (47.5%)	344/966 (35.6%)	377/1439 (26.2%)

^a^Age at the time of the blood cancer diagnosis.

^b^Analysis restricted to survivors in employment at the time of the blood cancer diagnosis.

^c^Chi^2^ test, p = 0.0001 for comparison of age groups.

^d^Chi^2^ test, p = 0.0031 for comparison of age groups.

^e^Chi^2^ test, p < 0.0001 for comparison of age groups.

#### Financial situation

Financial problems resulting from blood cancer-related income reduction were reported by 377 of 1439 responding survivors (26.2%), most often in survivors age 30–39 at the time of diagnosis (Table 3). The frequency of financial problems differed significantly among disease groups (p < 0.0001). They were most frequent in survivors from the AlloTx group (37.5% [194 of 517 responding patients]), followed by MM (31.4% [11/35]), aNHL/HL (23.1% [105/454]), MPN/CML (18.8% [19/101]), AML/ALL (17.9% [12/67]), iNHL/CLL (14.4% [35/243]), MGUS (5.9% [1/17]), and MDS (0.0% [0/5]). Survivors with a history of relapse more often reported financial problems than survivors without relapse (32.5% [98 of 304 responding survivors with known relapse] versus 25.7% [256/989]; p = 0.0298).

#### Health insurance

Health insurance was not significantly impacted by blood cancer (p = 0.8437). Both before and after blood cancer diagnosis, the most frequent type was statutory insurance (72.2% [1108 of 1535 responding survivors] versus 71.0% [1087/1532]), followed by statutory insurance with private supplementation (12.7% [195/1535] versus 13.6% [209/1532]), full private insurance (9.2% [141/1535] versus 9.1% [140/1532]), and state subsidy (5.6% [89/1535] versus 6.0% [92/1532]).

### Overall well-being and spirituality

#### Quality of life

**Figure 1:**
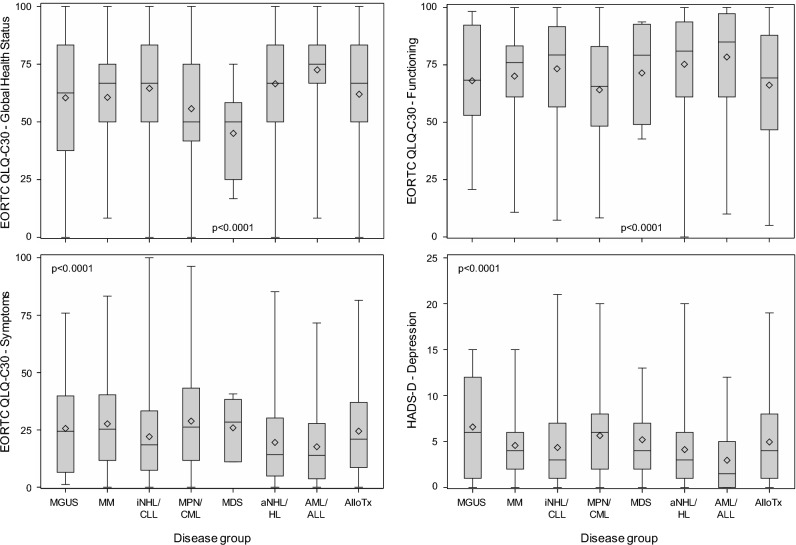
Quality of life in long-term survivors of different types of blood cancer at the time of the survey, as assessed by the EORTC QLQ C-30 (global health status, functioning, symptoms) and HADS questionnaires (depression). The results of the HADS anxiety scale (not shown) were similar to those of the depression scale. *MGUS* monoclonal gammopathy of undetermined significance, 16 responding survivors, *MM* multiple myeloma, 37 survivors, *iNHL/CLL* indolent non-Hodgkin lymphoma/chronic lymphocytic leukemia, 254 survivors, *MPN/CML* myeloproliferative neoplasm/chronic myeloid leukemia, 106 survivors, *MDS* myelodysplastic syndrome, 5 survivors, *aNHL/HL* aggressive non-Hodgkin lymphoma/Hodgkin lymphoma, 481 survivors, *AML/ALL* acute myeloid leukemia/acute lymphoblastic leukemia, 74 survivors, *AlloTx* allogeneic transplantation, 547 survivors. Box, range between the 25th and 75th percentile; whiskers, upper quartile with maximum and lower quartile with minimum, respectively; horizontal line, median; diamond, mean.

**Figure 2:**
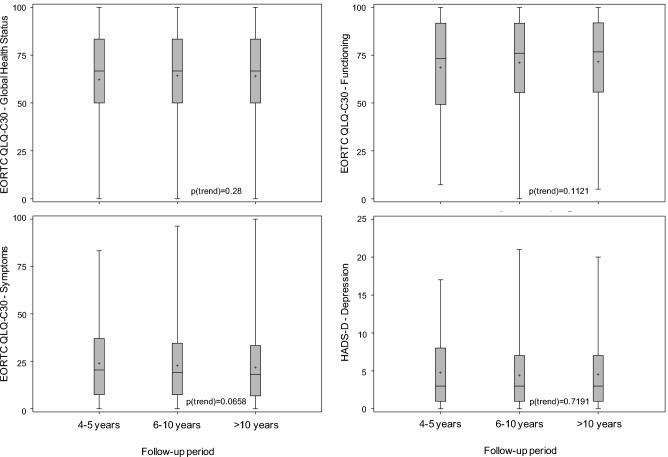
Quality of life in long-term blood cancer survivors at the time of the survey in relation to the duration of follow-up, as assessed by the EORTC QLQ C-30 (global health status, functioning, symptoms) and HADS questionnaires (depression). The results of the HADS anxiety scale (not shown) were similar to those of the depression scale. Follow-up 4–5 years, 284 responding survivors; 6–10 years, 516 survivors; > 10 years, 720 survivors. Box, range between the 25th and 75th percentile; whiskers, upper quartile with maximum and lower quartile with minimum, respectively; horizontal line, median; diamond, mean.

Quality of life significantly differed among disease groups, with best scores in AML/ALL, intermediate scores in aNHL/HL, iNHL/CLL, and AlloTx, and worst scores in MPN/CML (Fig. 1). Findings in MGUS, MM, and MDS were based on very small numbers. Quality of life was similar among survivors followed up for 4–5, 6–10, or > 10 years (Fig. 2).

#### Religion

On a scale from 0 (totally unimportant) to 4 (very important), the attitude towards religion showed a statistically non-significant trend (p = 0.0649) towards greater importance immediately after blood cancer treatment and during follow-up (Table 4).

**Table 4:** Attitude towards religion before the diagnosis of blood cancer, at the end of treatment, and at the time of the survey in 1292 survivors evaluating all three time-points.

	Number of survivors affected (% of total)		
	Before diagnosis	At end of treatment	At time of survey
Totally unimportant	242 (18.7%)	246 (19.0%)	246 (19.0%)
Rather unimportant	210 (16.3%)	177 (13.7%)	182 (14.1%)
Moderately important	424 (32.8%)	376 (29.1%)	387 (30.0%)
Quite important	233 (18.0%)	291 (22.5%)	270 (20.9%)
Very important	183 (14.2%)	202 (15.7%)	207 (16.0%)

Chi^2^ test, p = 0.0649.

## Discussion

The major results of this study are the following: first, blood cancer and its treatment appeared to have little impact on relationships with family members and friends. Second, employment was severely affected, with a substantial proportion of survivors discontinuing work permanently. Third, blood cancer-related financial problems were frequent. Fourth, quality of life differed significantly among disease groups.

In the ABC study, the proportion of married patients remained constant before and after blood cancer. In blood cancer survivors from France, Norway, and the USA, the divorce rate was slightly lower than in the general population^15,16,18^. Similar observations have been made for other types of cancer survivors^32^. The relationship with family members was described as unchanged by most ABC study participants. Improvements were reported more often than deteriorations in the relationship with children and in the relationship with partners who had witnessed the whole trajectory from blood cancer diagnosis to follow-up care at the time of the survey. This is in line with an early report on American Hodgkin lymphoma survivors where positive changes in family, friend and other relationships were more often encountered than negative changes^19^. In the ABC study, the number of social contacts was not influenced by blood cancer. In a French study restricted to Hodgkin lymphoma, loss of a friend was less often observed in study participants than in matched controls^15^.

Almost half of the patients reported a temporary impairment of their vocational activities, with an average period off work of 11 months. This is consistent with other reports where the average time off work was 5 months for all cancer patients combined^33^, 9 months for hematological patients in general^21^, and 14 months for patients undergoing autologous blood stem cell transplantation^23^. The same is true for the observation that more than a third of ABC study participants discontinued work permanently. The proportion of patients not returning to work has been reported to be 34–36% for all cancers combined^33,34^, 23–27% for hematological cancers in general^21,27^, 40% for allogeneic transplantation^35^, and 45–50% for autologous transplantation^23,30^. In a registry-based Danish study, the return-to-work rate was high in patients with lymphoma (72–89%), intermediate in leukemia (51–76%), and low in multiple myeloma (32%)^21^. Unemployment has repeatedly been identified as a risk factor for poor quality of life^25,30,35,36^.

The proportion of blood cancer survivors receiving a disability pension was lower in the ABC study (7.9%) than in other countries. In Denmark, the disability rate was 17% for hematological patients in general^22^ and 27% for patients undergoing autologous transplantation^23^. In the USA, it was 34% for all cancer patients combined^37^ and 39% for allogeneic transplant recipients^35^. The discrepancies may be related to differences in health care systems, follow-up periods, patient selection, and evaluation methods (questionnaire-, interview-, or registry-based studies). Distinguishing between disability and retirement pensions may have been difficult for ABC study participants. At the time of the survey, 38.1% of survivors received a retirement pension. In Denmark, early retirement was observed more frequently among patients with leukemia and non-Hodgkin lymphoma than among most other cancer patients^24^.

More than a quarter of ABC study participants reported financial problems resulting from blood cancer-related income reduction. The proportion was even higher among allogeneic transplant recipients and patients with relapsed disease. Our questionnaire did not allow us to specify the areas affected by financial problems. Expenditures for health care, significantly contributing to disease-related economic burden in the USA^37^, were unlikely to be of importance in ABC study participants, since in Germany the treatment costs are covered by a mandatory health insurance. For most participants, the type of insurance did not differ before and after blood cancer. In the Netherlands, financial problems were often encountered when lymphoma survivors attempted to obtain a life insurance (problems reported by 38–60% of survivors) or a property mortgage (63–73%)^28,29^. In the USA, the annual productivity loss unrelated to health care costs has been estimated to be $ 2250 for cancer patients below age 40 and $ 1018 for patients above age 40^37^. In Germany, a monthly loss of 500 € has been estimated for allogeneic transplant recipients^34^.

At a median follow-up of 9 years, quality of life was best in patients with a history of acute leukemia not treated by allogeneic transplantation. In an early interview-based investigation from the USA, acute leukemia survivors enjoyed better quality of life than Hodgkin lymphoma survivors^38^. The cure rate in acute leukemia is considerably lower than in Hodgkin lymphoma, but surviving patients are at much lower risk for late relapses, second primary malignancies, cardiovascular complications, and debilitating long-term treatment effects, such as polyneuropathy (unpublished results from the ABC study).

Unexpectedly, the worst quality-of-life scores were recorded in patients with myeloproliferative disorders. Although these diseases can only be cured by allogeneic transplantation, their prognosis is among the most favorable of all types of blood cancer. Long-term control of chronic myeloid leukemia is the rule, and the life expectancy of patients with myeloproliferative neoplasms approaches that of the general population^39^. Quality of life, however, has consistently been reported as low^40–43^. Unlike other types of blood cancer, myeloproliferative diseases are active during the follow-up period, and, in blood cancer survivors, active disease is associated with poor quality of life^44,45^. In addition, myeloproliferative diseases harbor a small risk of transformation to an almost invariably deadly type of acute leukemia which may be perceived as a distressing prospect. Most types of myeloproliferative diseases require continuous treatment that may be poorly tolerated. The patients’ major complaint, fatigue, reduces social interactions and promotes isolation^40,41,43,46,47^.

Because we did not have a matched control group, we were unable to compare the quality of life of ABC study participants with that of the general population^31^. Other studies have come to the conclusion that quality of life is lower in blood cancer survivors than in non-affected controls^28,29,35,36,42,43,48^. In the ABC study, the quality of life appeared to be independent of follow-up duration which is in line with earlier observations^35,36,45,49^.

Religion tended to become more important after the advent of blood cancer, although this finding did not reach statistical significance. Deepening of religious and spiritual beliefs after blood cancer treatment has previously been reported for allogeneic transplant recipients from the USA^50^.

Strengths and weaknesses of the ABC study have been discussed before^31^. In brief, limitations include participation and recall biases, inherent in any retrospective study, and a prevalence-incidence bias, i.e., restriction of the analysis to individuals alive at the time of the survey. In addition, MDS, MGUS, and MM were underrepresented^31^, precluding a meaningful comparison with other disease groups. Finally, the cross-sectional design of the ABC study prevented firm conclusions with regard to temporal changes^20^. Since the follow-up period spanned many years, the interval between the time before and after blood cancer varied from participant to participant. Restricting the study to survivors with an interval of at least 3 years since the last intense treatment, however, insured that the disease was well controlled, leaving a reasonable amount of time to adapt to the consequences of the disease and its treatment.

In conclusion, a large proportion of long-term blood cancer survivors reported a significant impairment of vocational activities, which was associated with reduced income and financial problems. Unemployment is a risk factor for poor quality of life^25,30,35,36^. Efforts should be made at an early stage to reestablish the patients’ ability to work.

### Supplementary Information

Supplementary Information.

## Data Availability

The datasets generated and analyzed during the current study are available from the corresponding author on reasonable request.
