# Supplementary material for: Impairment of vocational activities and financial problems are frequent among German blood cancer survivors
Source: Sci Rep. 2023 Dec 21;13:22856. doi: 10.1038/s41598-023-50289-9 (PMC10739705; doi:10.1038/s41598-023-50289-9)
Supplement: Supplementary file 1 — Supplementary Information. [file 41598_2023_50289_MOESM1_ESM.pdf]

Supplementary Information

Impairment of vocational activities and financial problems are frequent among German blood cancer survivors

Julia Baum,[1] Hildegard Lax,[2] Nils Lehmann,[2] Anja Merkel-Jens,[2] Dietrich W. Beelen,[3] Karl-Heinz Jöckel,[2] Ulrich Dührsen[1]

[1] Klinik für Hämatologie, Universitätsklinikum Essen, Universität Duisburg-Essen, Germany

[2] Institut für Medizinische Informatik, Biometrie und Epidemiologie, Universität Duisburg-Essen, Germany

[3] Klinik für Knochenmarktransplantation, Universitätsklinikum Essen, Universität Duisburg-Essen, Germany

Corresponding author:
Prof. Dr. Ulrich Dührsen
Klinik für Hämatologe
Universitätsklinikum Essen
Hufelandstraße 55
45147 Essen
Tel.: 02102-847374
Email: ulrich.duehrsen@uk-essen.de

Questions related to living conditions (original German version) page 2 - 9

Questions related to living conditions (English translation) page 10 - 17

Aftercare in blood cancer survivors (ABC study)

Retrospective part – Patient questionnaire

Questions related to living conditions

Original German version

Persönliche Lebenssituation

1. **Wo wohnten Sie vor erstmaliger Behandlung** (bei intensiv behandlungsbedürftigen Erkrankungen) **/ vor erstmaliger Feststellung Ihrer Blutkrebserkrankung** (bei nicht behandlungsbedürftigen oder dauerhaft mit Tabletten behandlungsbedürftigen Erkrankungen)**? Bitte geben Sie die Adresse an.**

   Postleitzahl und Wohnort: ____

   Straße und Hausnummer: ____

2. **Wie war Ihr Familienstand bei erstmaliger Feststellung der Blutkrebserkrankung und nach Beendigung der intensiven Behandlung und wie ist Ihr Familienstand heute?**
   (Wenn Ihre Erkrankung nicht intensiv behandelt wurde, lassen Sie die entsprechende Spalte bitte frei!)

	Bei erstmaliger Feststellung der Blutkrebserkrankung	Nach Beendigung der intensiven Behandlung	Heute
Ledig	☐1	☐1	☐1
Verheiratet/ Verpartnert	☐2	☐2	☐2
Geschieden	☐3	☐3	☐3
Getrennt lebend	☐4	☐4	☐4
Verwitwet	☐5	☐5	☐5

3. **Lebten / leben Sie mit einem festen Partner / einer festen Partnerin zusammen?**

	Bei erstmaliger Feststellung der Blutkrebserkrankung		Heute
Nein	☐1	Nein	☐1

Ja	☐2	Ja, mit demselben Partner / derselben Partnerin	☐2
		Ja, mit einem neuen Partner / einer neuen Partnerin	☐3

4. Wenn Sie noch mit demselben Partner / derselben Partnerin zusammenleben wie vor der Erkrankung, hat sich die Beziehung zu Ihrem Partner / Ihrer Partnerin durch die Erkrankung verändert?

☐1 Verbessert
☐2 Unverändert
☐3 Verschlechtert
☐4 Die Frage trifft auf mich nicht zu, da ich nicht mit demselben Partner / derselben Partnerin zusammenlebe wie vor der Erkrankung

5. Was hat sich in der Beziehung zu Ihrem Partner / Ihrer Partnerin verändert? Bitte beschreiben Sie dies mit Ihren eigenen Worten.

____

____

____

6. Wenn Sie ein Kind bzw. Kinder haben, hat sich die Beziehung zu Ihrem Kind / Ihren Kindern durch die Erkrankung verändert?

☐1 Verbessert
☐2 Unverändert
☐3 Verschlechtert
☐4 Die Frage trifft auf mich nicht zu, da ich keine Kinder habe

7. Was hat sich in der Beziehung zu Ihrem Kind / Ihren Kindern verändert? Bitte beschreiben Sie dies mit Ihren eigenen Worten.

____

____

____

8. **Wie wichtig war / ist Ihnen Religion und / oder die Beschäftigung mit geistigen Dingen?** (Wenn Ihre Erkrankung nicht intensiv behandelt wurde, lassen Sie die entsprechende Spalte bitte frei!)

	Bei erstmaliger Feststellung der Blutkrebserkrankung	Nach Beendigung der intensiven Behandlung	Heute
Ganz unwichtig	☐1	☐1	☐1
Ziemlich unwichtig	☐2	☐2	☐2
Mäßig wichtig	☐3	☐3	☐3
Ziemlich wichtig	☐4	☐4	☐4
Sehr wichtig	☐5	☐5	☐5

9. **Gibt / gab es Krebserkrankungen in Ihrer Familie?**

	Ja	Nein	Weiß ich nicht
Ehe- / Lebenspartner / -in	☐1	☐2	☐3
Eltern	☐1	☐2	☐3
Geschwister	☐1	☐2	☐3
Kinder	☐1	☐2	☐3
Sonstige Haushaltsangehörige<br>Wer? (bitte eintragen)	☐1	☐2	☐3

Wenn ja, welche Person ist / war an welcher Erkrankung erkrankt?

Krankenversicherungsstatus

10. Wie waren / sind Sie krankenversichert?
(Hier sind mehrere Antworten möglich!)

	Bei erstmaliger Feststellung der Blutkrebserkrankung	Heute
Gesetzlich krankenversichert	☐1	☐1
Ausschließlich privat versichert	☐2	☐2
Private Zusatzversicherung	☐3	☐3
Beihilfeberechtigt	☐4	☐4
Sonstiges (bitte angeben)	☐5	☐5

11. Bitte nennen Sie den Name Ihrer Krankenversicherung bei erstmaliger Feststellung der Blutkrebserkrankung und heute.

Krankenversicherung bei erstmaliger Feststellung der Blutkrebserkrankung	Krankenversicherung heute

Berufliche Situation und Erwerbsfähigkeit

12. Welchen höchsten Schulabschluss haben Sie?

☐1 Ich bin noch Schüler
☐2 Ich habe die Schule ohne Abschluss beendet
☐3 Hauptschule / Volksschulabschluss
☐4 Realschule / mittlere Reife
☐5 Polytechnische Oberschule
☐6 Fachhochschulreife
☐7 Abitur / allgemeine Hochschulreife
☐8 Abitur über den zweiten Bildungsweg
☐9 Anderer Schulabschluss,
und zwar (bitte angeben): ___________________________________

13. Welches ist Ihr höchster berufsbildender Abschluss?

- ☐01 Ich bin noch in beruflicher Ausbildung / Schüler
- ☐02 Keinen beruflichen Abschluss und derzeit nicht in Ausbildung
- ☐03 Beruflich-betriebliche Ausbildung (Lehre)
- ☐04 Beruflich-schulische Ausbildung (z. B. Handelsschule)
- ☐05 Ausbildung an einer Fachschule der DDR
- ☐06 Fach- / Techniker- / Meisterschule / Berufsakademie
- ☐07 Bachelor an (Fach-)Hochschule
- ☐08 Fachhochschulabschluss (z. B. Diplom, Master)
- ☐09 Universitätsabschluss (z. B. Diplom, Master, Magister, Staatsexamen)
- ☐10 Promotion
- ☐11 Anderer beruflicher Abschluss,
  und zwar (bitte angeben): ____

14. Waren / sind Sie berufstätig / erwerbstätig?

	Bei erstmaliger Feststellung der Blutkrebserkrankung	Heute
Vollzeit erwerbstätig	☐01	☐01
Mindestens halbtags erwerbstätig	☐02	☐02
Weniger als halbtags erwerbstätig	☐03	☐03
Altersteilzeit	☐04	☐04
Geringfügig beschäftigt (400 Euro- oder Mini-Job)	☐05	☐05
„Ein-Euro-Job“ (bei Bezug von Arbeitslosengeld II)	☐06	☐06
Gelegentlich oder unregelmäßig beschäftigt	☐07	☐07
In einer beruflichen Ausbildung / Lehre	☐08	☐08
In Umschulung	☐09	☐09
Bundesfreiwilligendienst, freiwilliges, soziales / ökologisches Jahr	☐10	☐10
Mutterschutz, Erziehungsurlaub, Elternzeit oder sonstige Beurlaubung	☐11	☐11
Nicht erwerbstätig	☐12	☐12

15. Wenn Sie nicht erwerbstätig waren / sind, welche der folgenden Angaben traf / trifft dann auf Ihre Situation zu?

	Bei erstmaliger Feststellung der Blutkrebserkrankung	Heute
Schüler / -in an einer allgemeinbildenden Schule	☐1	☐1
Student / -in	☐2	☐2
Rentner / -in, Pensionär / -in, in Vorruhestand	☐3	☐3
Arbeitslos	☐4	☐4
Dauerhaft erwerbsunfähig	☐5	☐5
Hausfrau / Hausmann	☐6	☐6
Sonstiges (bitte angeben)	☐7	☐7

16. Welchen Beruf haben Sie ausgeübt? Welchen Beruf üben Sie gegenwärtig aus?

Bei erstmaliger Feststellung der Blutkrebserkrankung	Heute

17. Zu welcher Gruppe gehörte / gehört ihr Beruf?

	Bei erstmaliger Feststellung der Blutkrebserkrankung	Heute
Mithelfende / -r Familienangehörige / -r	☐1	☐1
Ausbildung	☐2	☐2
Arbeiter / -in	☐3	☐3
Angestellte / -r	☐4	☐4
Beamter / Beamtin, Richter / -in, Berufssoldat / -in	☐5	☐5
Selbständige / -r im Handel, im Gewerbe, im Handwerk, in der Industrie, in der Dienstleistung	☐6	☐6

Akademiker / -in in freiem Beruf<br>(Arzt / Ärztin, Rechtsanwalt / -anwältin, Steuerberater / -in u. ä.)	☐7	☐7
Selbständige / -r Landwirt / -in	☐8	☐8

18. Bitte geben Sie an, wie lange Sie nach erstmaliger Behandlungsbedürftigkeit (bei intensiv behandlungsbedürftigen Erkrankungen) **/ nach erstmaliger Feststellung Ihrer Blutkrebserkrankung** (bei nicht behandlungsbedürftigen oder dauerhaft mit Tabletten behandlungsbedürftigen Erkrankungen**) nicht aktiv am Berufsleben teilgenommen haben** (Krankschreibung, Berufsunfähigkeit, Erwerbsunfähigkeit).

☐1 Ich habe stets aktiv am Berufsleben teilgenommen
☐2 Ich konnte für eine bestimmte Zeit nicht aktiv am Berufsleben teilhaben, und zwar für (bitte angeben):
_____________ Monate; ___________ Jahre
☐3 Ich nehme bis zum heutigen Tag nicht wieder aktiv am Berufsleben teil, und zwar seit (bitte angeben):
____________ Monaten; __________ Jahren
☐4 Die Frage trifft auf mich nicht zu, da ich bereits vor erstmaliger Behandlungsbedürftigkeit bzw. erstmaliger Feststellung der Blutkrebs-erkrankung keine berufliche Tätigkeit wahrgenommen habe

19. Bitte geben Sie an, wie lange es nach erstmaliger Behandlungsbedürftigkeit (bei intensiv behandlungsbedürftigen Erkrankungen) **/ nach erstmaliger Feststellung Ihrer Blutkrebserkrankung** (bei nicht behandlungsbedürftigen oder dauerhaft mit Tabletten behandlungsbedürftigen Erkrankungen) **gedauert hat, bis Sie im gleichen Maße am Berufsleben teilgenommen haben wie vor erstmaliger Behandlungsbedürftigkeit bzw. vor erstmaliger Feststellung der Erkrankung.**

☐1 Meine berufliche Aktivität war nicht eingeschränkt
☐2 Ich habe die gleiche berufliche Aktivität zurück erlangt, und zwar nach (bitte angeben):
_________ Monaten; ____________ Jahren
☐3 Ich habe meine berufliche Aktivität bis zum heutigen Tag nicht im früheren Maße wiedererlangt,
und zwar seit (bitte angeben): ___________ Monaten; ___________ Jahren
☐4 Die Frage trifft auf mich nicht zu, da ich bereits vor erstmaliger Behandlungsbedürftigkeit bzw. erstmaliger Feststellung der Blutkrebs-erkrankung keine berufliche Tätigkeit wahrgenommen habe

20. Ist es durch die Blutkrebserkrankung zu einem Einkommensausfall gekommen, der Sie in finanzielle Schwierigkeiten gebracht hat?

☐1 Ja
☐2 Nein

Soziale Kontakte

21. Bitte geben Sie in der nachfolgen Tabelle an, welche sozialen Kontakte Sie vor erstmaliger Behandlungsbedürftigkeit (bei intensiv behandlungsbedürftigen Erkrankungen) / **nach erstmaliger Feststellung Ihrer Blutkrebserkrankung** (bei nicht behandlungsbedürftigen oder dauerhaft mit Tabletten behandlungsbedürftigen Erkrankungen) **hatten.**

	Vor erstmaliger Behandlung / bei erstmaliger Feststellung der Blutkrebserkrankung	Heute
Wie viele lebende Kinder (leibliche, Stief- und Adoptivkinder) hatten / haben Sie?		
Wie viele Kinder sahen / sehen Sie wenigstens einmal im Monat?		
Wie viele Verwandte hatten / haben Sie, denen Sie sehr nahe standen / stehen?		
Wie viele dieser nahe stehenden Verwandten sahen / sehen Sie wenigstens einmal im Monat?		
Ganz allgemein, wie viele wirklich enge Freunde hatten / haben Sie?		
Wie viele von diesen engen Freunden sahen / sehen Sie wenigstens einmal im Monat?		

Aftercare in blood cancer survivors (ABC study)

Retrospective part – Patient questionnaire

Questions related to living conditions

English translation

Personal living situation

1. **Where did you live before your first treatment** (for diseases requiring intensive treatment) / **before your blood cancer was diagnosed for the first time** (for diseases not requiring treatment or requiring long-term treatment with tablets)? **Please enter the address.**

   Zip code and place of residence: ______________________

   Street and house number: ______________________

2. **What was your marital status when you were first diagnosed with blood cancer and after completing intensive treatment, and what is your marital status now?** (If your disease was not treated intensively, please leave the corresponding column blank).

	When first diagnosed with blood cancer	After completion of intensive treatment	Today
Single	☐1	☐1	☐1
Married / partnered	☐2	☐2	☐2
Divorced	☐3	☐3	☐3
Separated	☐4	☐4	☐4
Widowed	☐5	☐5	☐5

3. **Did you / do you live with a steady partner?**

	When first diagnosed with blood cancer		Today
No	☐1	No	☐1
Yes	☐2	Yes, with the same partner	☐2
		Yes, with a new partner	☐3

4. If you are still living with the same partner as before the illness, has the relationship with your partner changed as a result of the illness?

☐1 Improved
☐2 Unchanged
☐3 Deteriorated
☐4 This question does not apply to me because I do not live with the same partner as before the illness

5. What has changed in your relationship with your partner? Please describe this in your own words.

____

____

____

6. If you have a child or children, has the relationship with your child/children changed as a result of the illness?

☐1 Improved
☐2 Unchanged
☐3 Deteriorated
☐4 This question does not apply to me as I do not have any children

7. What has changed in your relationship with your child/children? Please describe this in your own words.

____

____

____

8. **How important were / are religion and/or spiritual matters to you?** (If your disease was not treated intensively, please leave the corresponding column blank).

	When first diagnosed with blood cancer	After completion of intensive treatment	Today
Totally unimportant	☐1	☐1	☐1
Rather unimportant	☐2	☐2	☐2
Moderately important	☐3	☐3	☐3
Quite important	☐4	☐4	☐4
Very important	☐5	☐5	☐5

9. **Is/was there a history of cancer in your family?**

	Yes	No	Don't know
Spouse / partner	☐1	☐2	☐3
Parents	☐1	☐2	☐3
Siblings	☐1	☐2	☐3
Children	☐1	☐2	☐3
Other household members	☐1	☐2	☐3
Who? (please enter)			

If yes, which person is / was ill with which illness?

____

____

____

Health insurance status

10. How were / are you insured?
(Several answers are possible here!)

	When first diagnosed with blood cancer	Today
Statutory health insurance	☐1	☐1
Exclusively privately insured	☐2	☐2
Private supplementary insurance	☐3	☐3
Entitled to benefits (state subsidy)	☐4	☐4
Other<br>(please specify)	☐5	☐5

11. Please state the name of your health insurance when you were first diagnosed with blood cancer and today.

Health insurance when the blood cancer was first diagnosed	Health insurance today

Professional situation and earning capacity

12. What is your highest school-leaving qualification?

☐1 I am still a pupil
☐2 I finished school without any qualifications
☐3 Secondary school / elementary school certificate
☐4 Realschule / intermediate school leaving certificate
☐5 Polytechnic secondary school
☐6 Advanced technical college certificate
☐7 Abitur / general higher education entrance qualification
☐8 Abitur via second educational path
☐9 Other school-leaving qualification,
namely (please specify): ____

13. What is your highest vocational qualification?

- ☐01 I am still in vocational training / student
- ☐02 No vocational qualification and currently not in training
- ☐03 Vocational training (apprenticeship)
- ☐04 Vocational school education (e.g. commercial school)
- ☐05 Training at a technical college in the German Democratic Republic
- ☐06 Technical / technician / master craftsman school / vocational academy
- ☐07 Bachelor's degree from a (technical) university
- ☐08 Degree from a university of applied sciences (e.g. diploma, master's degree)
- ☐09 University degree (e.g. Diplom, Master's, Magister, state examination)
- ☐10 Doctorate
- ☐11 Other professional qualification,
  namely (please specify): ____

14. Were / are you employed / gainfully employed?

	When first diagnosed with blood cancer	Today
Full-time employment	☐01	☐01
Working at least half-time	☐02	☐02
Less than half-time employment	☐03	☐03
Partial retirement	☐04	☐04
Marginally employed (400 euro or mini-job)	☐05	☐05
"One-euro job" (in receipt of unemployment benefit II)	☐06	☐06
Occasionally or irregularly employed	☐07	☐07
In vocational training / apprenticeship	☐08	☐08
In retraining	☐09	☐09
Federal voluntary service, voluntary social / ecological year	☐10	☐10
Maternity protection, parental leave, parental leave or other leave of absence	☐11	☐11
Not gainfully employed	☐12	☐12

15. If you were/are not gainfully employed, which of the following applied/applies to your situation?

	When first diagnosed with blood cancer	Today
Student at a general education school	☐1	☐1
Student	☐2	☐2
Pensioner, retired, in early retirement	☐3	☐3
Unemployed	☐4	☐4
Permanently unable to work	☐5	☐5
Housewife / househusband	☐6	☐6
Other<br>(please specify)	☐7	☐7

16. What was your previous occupation? What is your current occupation?

When first diagnosed with blood cancer	Today

17. Which group did / does your profession belong to?

	When first diagnosed with blood cancer	Today
Contributing family member	☐1	☐1
Apprenticeship	☐2	☐2
Worker	☐3	☐3
Employee	☐4	☐4
Civil servant, judge, professional soldier	☐5	☐5
Self-employed person in commerce, trade, crafts, industry, or service sector	☐6	☐6
Academic in a liberal profession (physician, lawyer, tax consultant, etc.)	☐7	☐7
Self-employed farmer	☐8	☐8

18. **Please state how long you have not actively participated in working life** (sick leave, incapacity to work, disability) **after first needing treatment** (for illnesses requiring intensive treatment) **/ after first being diagnosed with blood cancer** (for illnesses not requiring treatment or requiring long-term treatment with tablets)**.**

- ☐1 I have always actively participated in working life
- ☐2 I was unable to actively participate in working life for a certain period of time, for (please specify):
  ____________ months; __________ years
- ☐3 I have not been actively participating in working life again to this day, for (please specify):
  ____________ months; __________ years
- ☐4 The question does not apply to me, as I did not carry out any professional activity when I first needed treatment for blood cancer / when I was first diagnosed with blood cancer

19. **Please indicate how long it took after first needing treatment** (for diseases requiring intensive treatment) **/ after first diagnosis of your blood cancer** (for diseases not requiring treatment or requiring long-term treatment with tablets) **before you were able to participate in your professional life to the same extent as before first treatment or diagnosis of the disease, respectively.**

- ☐1 My professional activity was not restricted
- ☐2 I have regained the same level of professional activity, after (please specify):
  __________ months; __________ years
- ☐3 To this day, I have not regained my occupational activity to the same extent as before,
  for (please specify): __________ months; __________ years
- ☐4 This question does not apply to me, as I was already disabled before first treatment or diagnosis of the disease, respectively

20. **Has the blood cancer resulted in a loss of income that has caused you financial difficulties?**

- ☐1 Yes
- ☐2 No

Social contacts

21. **Please indicate in the table below which social contacts you had before you first needed treatment** (for diseases requiring intensive treatment) **/ after your blood cancer was diagnosed for the first time** (for diseases not requiring treatment or requiring long-term treatment with tablets)**.**

	Before first treatment / after first diagnosis of blood cancer	Today
How many living children (biological, stepchildren and adopted children) did / do you have?		
How many children did / do you see at least once a month?		
How many relatives did / do you have with whom you were / are very close?		
How many of these close relatives did / do you see at least once a month?		
In general, how many really close friends did / do you have?		
How many of these close friends did / do you see at least once a month?		
